# Effect of thickness and reaction media on properties of ZnO thin films by SILAR

**DOI:** 10.1038/s41598-022-04782-2

**Published:** 2022-01-17

**Authors:** Gani Yergaliuly, Baktiyar Soltabayev, Sandugash Kalybekkyzy, Zhumabay Bakenov, Almagul Mentbayeva

**Affiliations:** 1 Department of Chemical and Material Engineering, School of Engineering and Digital Sciences, Nazarbayev University, Nur-Sultan, 010000 Kazakhstan; 2 L.N. Gumilyov Eurasian National University, Nur-Sultan, 010000 Kazakhstan; 3 National Laboratory Astana, Nazarbayev University, Nur-Sultan, 010000 Kazakhstan

**Keywords:** Materials for optics, Nanoscale materials

## Abstract

Zinc oxide (ZnO) is one of the most promising metal oxide semiconductor materials, particularly for optical and gas sensing applications. The influence of thickness and solvent on various features of ZnO thin films deposited at ambient temperature and barometric pressure by the sequential ionic layer adsorption and reaction method (SILAR) was carefully studied in this work. Ethanol and distilled water (DW) were alternatively used as a solvent for preparation of ZnO precursor solution. Superficial morphology, crystallite structure, optical and electrical characteristics of the thin films of various thickness are examined applying X-ray diffraction (XRD) system, scanning electron microscopy, the atomic force microscopy, X-ray photoelectron spectroscopy, ultraviolet–visible spectroscopy, photoluminescence spectroscopy, Hall effect measurement analysis and UV response study. XRD analysis confirmed that thin films fabricated using ethanol or DW precursor solvents are hexagonal wurtzite ZnO with a preferred growth orientation (002). Furthermore, it was found that thin films made using ethanol are as highly crystalline as thin films made using DW. ZnO thin films prepared using aqueous solutions possess high optical band gaps. However, films prepared with ethanol solvent have low resistivity (10^–2^ Ω cm) and high electron mobility (750 cm^2^/Vs). The ethanol solvent-based SILAR method opens opportunities to synthase high quality ZnO thin films for various potential applications.

## Introduction

Different thin film fabrication methods such as spin coating^1^, sol–gel^2^, spray pyrolysis^3^, electro deposition^4^, chemical bath deposition^5^, magnetron sputtering^6^ and successive ion layer adsorption reaction (SILAR)^7^ etc. have been applied to produce zinc oxide (ZnO) layers. As compared to other deposition methods, SILAR has attractive advantages such as simplicity, reproducibility, environmentally friendliness, cost-effectiveness etc. SILAR is based on immersion of the substrate into separately placed cations and anions follows by rinsing after each reaction, which enables a heterogeneous reaction between the solid phase and the solvated ions in the solution. The SILAR is an ideal method for making uniform, compact and crystalline thin films^8^. The key privilege of the SILAR techniques is the comparatively high rate of layer growth, which is readily controlled by changing the adsorption and response time^9^. The technique has several distinctive capabilities: low immersion temperature, which avoids contamination, diffusion and redistribution of additional impurities^10^.

ZnO thin films along with other transition metal oxides such as In_2_O_3_, SnO_2_, TiO_2_ and WO_3_ are attracting considerable attention due to their high temperature stability and tunable electronic and phonon transport properties^11^. The results of many previous studies show that reaction media properties are critical for the evolution of ZnO nanostructures with well-defined morphology^12–15^. Furthermore, previous studies also indicated that the surface morphologies of thin films strongly influence the phonon path length, carrier concentration, and mobility^11^. The phonon path length is the path length for phonons propagating through nanostructures which is primarily used to describe lattice thermal conductivity^16^. This implies the importance of controlling the morphology (nanorods, plates, spindle-like, flowers, etc.) of materials to improve their thermoelectric properties. Recent studies have shown that ZnO thin films exhibit the highest thermoelectric power factor, up to 1 mW/mK^2^, among transition metal oxides^17^. Thus, ZnO nanostructures of various morphologies are attracting increased attention.

By this time, ZnO of various nanostructured morphologies, such as nanorods, spindle-like, nano-flowers were prepared using SILAR. In recent study by Abdulrahman et al.^18^, efforts were made to create a multifunctional ultraviolet (UV) detector based on ZnO nanorods. Desai et al.^19^ demonstrated a simple and effective method for growing well-oriented thin films of ZnO nanorods without any help of the seed layer. In the article^20^, authors investigated the wettability of flower-like ZnO films that showed hydrophobic behavior. Further, gas sensing properties of a flower-like ZnO was investigated and showed excellent performance^21^. The morphologies and resulting properties are accurately controlled by the reaction parameters, such as concentration, temperature, pH, and deposition cycle number^22,23^. The influence of the immersion cycle on structural, electrical, gas-sensitive, and other properties of thin ZnO layers is discussed in numerous works^24–27^. In the SILAR technique, in addition to the above discussed, precursors and annealing temperature play crucial roles. Ravichandran et al.^28^, investigated the effect of three different common precursors and two different annealing conditions on the structural and transparent conductive characteristic of synthesized ZnO films. Nevertheless, in most of these works ZnO thin films were formed in aqueous media, and mainly water-soluble precursors were utilized. At the same time, a number of precursor compounds are purely soluble in water. Therefore, it is crucial to study the ZnO film growth by SILAR in non-aqueous media.

There are a few studies^29–31^, where solvents such as ethylene, isopropyl alcohol, etc. were used in the process of obtaining ZnO by SILAR. The studies have shown that tin oxide and zinc oxide thin films by spray pyrolysis using alcohol solvents have a higher optical transmission and band gap compared to aqueous solvents due to better crystallinity and defect levels formed in the band gap^32–34^. Here, to our knowledge for the first time, we report the construction of ZnO thin films on glass substrates by SILAR, where pure ethanol was utilized as a solvent for precursors. Importantly, we compared the functionality of ZnO films grown from aqueous and alcohol solutions. Thereby, new findings on thickness and precursor solvent’s influence on the surface morphology, composition, optical and electrical properties of the deposited films are reported.

## Experimental part

### ZnO thin film growth

**Figure 1:**
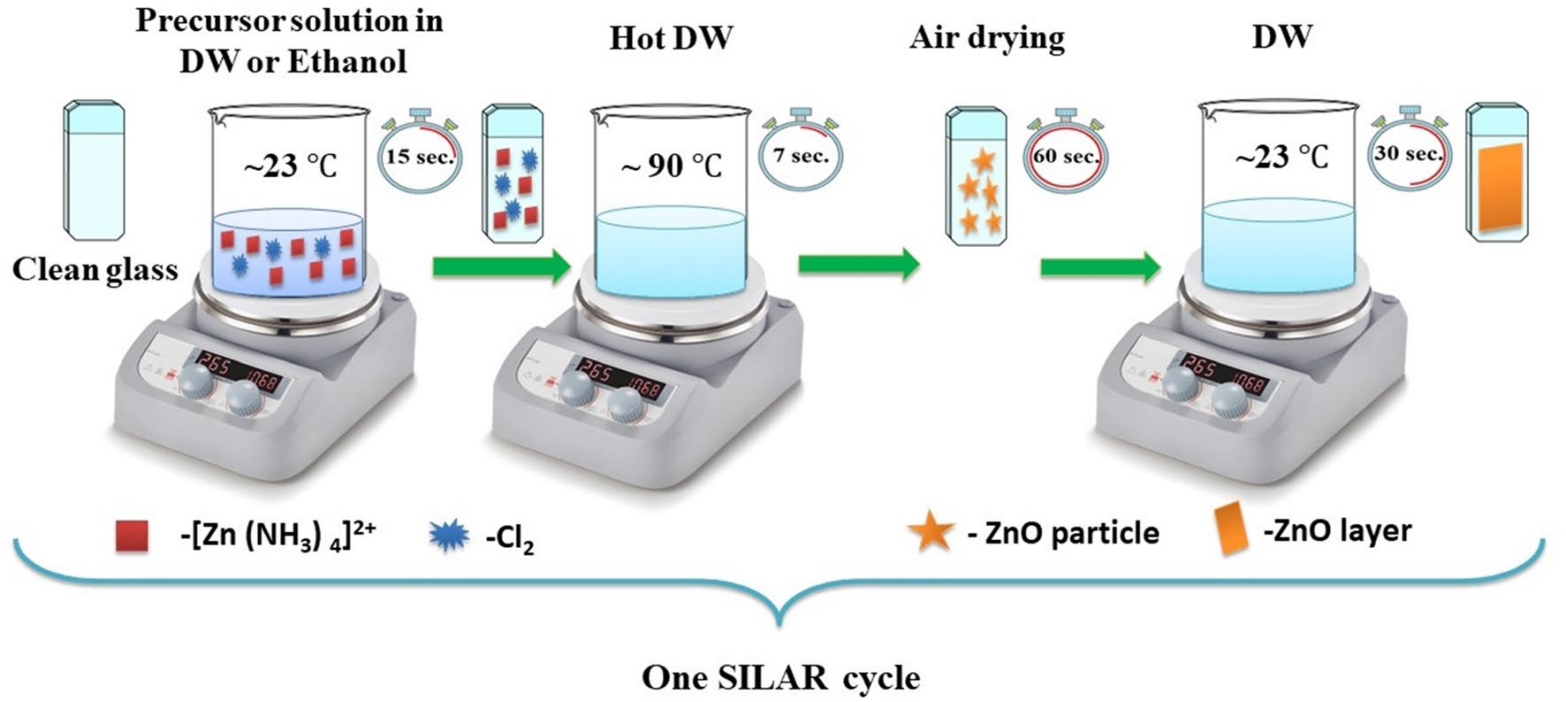
Scheme of synthesis of the ZnO thin films using the SILAR method.

ZnO films were fabricated on glass substrate using the SILAR method at room temperature and atmospheric pressure. Glass substrates were cleaned with an ultrasonic bath (in soap water, then distilled supply, and then in water: ethanol mixture with a mass ratio of 1:1) and were arid within nitrogen environment for half an hour. The solution for SILAR deposition was prepared by dissolving 1.363 g of Zinc chloride (ZnCl_2_, Sigma Aldrich purity ≥ 98%) in 100 mL distilled water (DW) or purified ethanol by adding ammonium hydroxide solution (25–30 wt% NH_4_OH, Sigma Aldrich) dropwise to form amino complex of zinc ([Zn(NH_3_)_4_]^2+^) until the pH of solutions was adjusted to $\sim$ 10.5. This solution was stirred for 30 min at room temperature for obtaining a homogenous solution. For deposition of ZnO thin film by SILAR, the following steps were conducted. First, glass substrate was immersed in the prepared solution for 15 s, then in hot water (90 °C) for 7 s. After, substrates were dried in the air environment for 60 s. Thus, one SILAR cycle of ZnO deposition was completed. The scheme of synthesis of the ZnO thin films for one SILAR cycle is shown in Fig. 1. The adsorption, reaction and rinsing times were chosen experimentally so that deposition occurred layerwise and resulted in homogeneous thin film structure. Films were deposited by repeating SILAR cycles 30, 40, 50 and 60 times. Deposited ZnO thin films grown in DW (ZnO-DW) and ethanol (ZnO-Eth) were named as ZnO-30 (DW or Eth), ZnO-40 (DW or Eth), ZnO-50 (DW or Eth) and ZnO-60 (DW or Eth), respectively. Further, the samples were annealed in an N_2_ environment at 500 ℃ for 2 h to obtain films with the ZnO crystalline phase. During the growth of the film, the following chemical reactions occur^35^:

1 $$ {\text{ZnCl}}_{{2}} + {\text{ 2NH}}_{{4}} {\text{OH }} \leftrightarrow {\text{ Zn}}\left( {{\text{OH}}} \right)_{{2}} + {\text{ 2NH}}_{{4}}^{ + } {\text{2Cl}}^{ - } $$

2 $$ {\text{Zn}}\left( {{\text{OH}}} \right)_{{2}} + {\text{ 4NH}}_{{4}}^{ + } \leftrightarrow \, [{\text{Zn}}({\text{NH}}_{{3}} )_{{4}} ]^{{{2} + }} + {\text{ 2H}}_{{2}} {\text{O }} + {\text{ 2H}}^{ + } $$

3 $$ \left[ {{\text{Zn}}\left( {{\text{NH}}_{{3}} } \right)_{{4}} } \right]^{{{2} + }} + {\text{ 4H}}_{{2}} {\text{O }} \to {\text{ Zn}}\left( {{\text{OH}}} \right)_{{2}} + {\text{ 4NH}}_{{4}}^{ + } + {\text{ 2OH}}^{ - } $$

4 $$ {\text{Zn}}\left( {{\text{OH}}} \right)_{{2}} \to {\text{ ZnO }} + {\text{ H}}_{{2}} {\text{O}} $$

The detailed procedure for applying thin films by SILAR was described in our previous study^36^. The thickness of prepared ZnO films were measured by spectroscopic ellipsometry where in the incident angle was fixed at 70 °C and the wavelength region from 330 to 1100 nm was scanned with 0.5 nm steps.

### Characterization techniques

The structural qualities of ZnO films were examined using the SmartLAB Rigaku X-ray diffraction (XRD) system (using Cu Kα λ = 1.5406 Å radiation). The morphological properties of the surface and elemental constitution of the samples were observed by using a Zeiss Crossbeam 540 scanning electronic microscope (SEM). The x-ray photoelectron spectroscopy (XPS) analysis was carried out with the Nexsa XPS system (Thermo Scientific). The atomic force microscope (AFM) SMART SPM 1000 was used to study the morphology and root mean square (RMS) distribution of particles. Metering of ZnO sample thickness was carried out by SEN research 4.0 ellipsometer device. The electrical parameters of the samples were derived via Hall measurement system (HMS-5500) at ambient temperature. Optical qualities of thin films were considered via ultraviolet–visible (UV–Vis) spectrophotometer (Evolution 3000 by Thermo Fisher) within the wavelength range 330–600 nm. Photoluminescence (PL) emission from the samples was collected by using a spectrofluorometric system (C9920-02, Hamamatsu Photonics K.K.) with a 350 nm laser source at room temperature. The transmission spectra were obtained using a spectrophotometer (The evolution 300 UV–Vis spectrophotometer, Thermo Scientific). Direct-Q Millipore filtration system was used for producing distilled water.

## Results and discussions

### Structural properties

**Figure 2:**
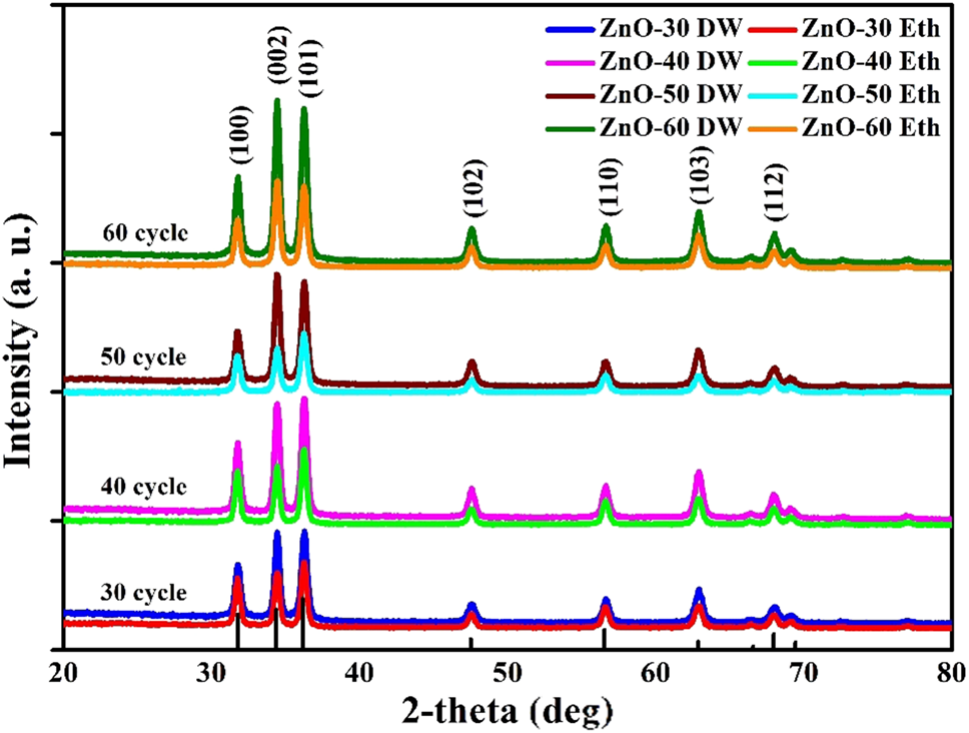
XRD patterns of ZnO thin films with different SILAR cycle.

To elaborate on the impact of thickness and reaction media on growth of zinc oxide nanostructures, XRD analysis of deposited ZnO thin films on the microscope glass was examined. Figure 2 shows the patterns of ZnO thin films with various deposition cycles (30, 40, 50 and 60) which were grown using distilled water and ethanol solvents.

All ZnO thin films exhibited high crystallinity with sharp characteristic peaks, where the presence of (100), (002) and (101) planes match to the hexagonal wurtzite crystal structure of ZnO. Furthermore, the increase of intensity preferably on the c-axis orientation along (002) plane by thickness growth was observed in cases of both water and alcohol media^37^. This intensity growth trend is in well agreement with the published works^19,38^, and also with ICSD card PDF-2 files (No: 03-065-0726).

It is worth to note that in both cases, samples with a deposition cycle of 50 and 60 showed the highest intensity of the diffraction peak. This indicates that the crystallinity quality of ZnO films cultivated with an increase of cycle number (see Table 1), which was also discussed in previously published studies^39,40^. The crystal sizes (D) of ZnO structures were appreciated by using the well-known of Debye–Scherrer equation^41^ as follows:

5 $$D=\frac{0.94\lambda }{\beta cos\theta }$$

where $\lambda$ is the wavelength of the incident X-ray ($\lambda =1.5406 \AA$), $\beta$ is the full width at half maximum (FWHM), and $\theta$ is the diffraction angle at which the peak of a particular orientation occurs. The dislocation density (δ) is representing the size of the defect in the crystallite. The higher δ values specify inferior crystallinity levels for the films because δ inversely proportional to the square of crystal size^42^:

6 $$\delta =\frac{1}{{D}^{2}}$$

The interplanar distance “$d$”, lattice constants $(a=b,c)$ and lattice strains ($\varepsilon$) for the wurtzite hexagonal structure of ZnO were computed using the following equations^43^:

7 $$\frac{1}{{d}^{2}}=\frac{4}{3}\left(\frac{{h}^{2}+hk+{k}^{2}}{{a}^{2}}\right)+\frac{{l}^{2}}{{c}^{2}}$$

8 $$\varepsilon =\frac{\beta }{4tan\theta }$$

where $h,k \mathrm{and} l$ are the Miller indices of the plane. From Table 1 can be seen the calculated lattice parameters, strains, interplanar distance, the intensity, and FWHM of the peaks’ values of the films.

**Table 1:** Influence of SILAR cycles number on the crystallite size (D), FWHM, dislocation density (δ), lattice strain (ε), interplanar distance (d) and lattice constants (a = b and c) of ZnO films along diffraction peak (002).

Sample name	2θ (°)	D (nm)	FWHM	δ (Å)^-2^ × 10^–6^	ε (10^–3^)	d (Å)	Lattice constant (Å)		Thickness (nm)
							a = b	c	
**DW**									
ZnO-30	34.419	22.720	0.36952	19.372	11.81	2.603	3.0062	5.2069	192 ± 7
ZnO-40	34.407	23.182	0.36216	18.608	11.31	2.604	3.0072	5.2087	217 ± 9
ZnO-50	34.423	20.502	0.40951	23.791	10.66	2.603	3.0058	5.2063	329 ± 12
ZnO-60	34.420	21.395	0.39242	21.846	10.45	2.603	3.0061	5.2067	498 ± 16
**Ethanol**									
ZnO-30	34.440	23.289	0.3605	18.437	10.62	2.602	3.0079	5.2040	136 ± 6
ZnO-40	34.437	23.191	0.3620	18.593	10.43	2.602	3.0048	5.2044	191 ± 9
ZnO-50	34.445	22.773	0.3687	19.282	10.39	2.601	3.0041	5.2033	284 ± 9
ZnO-60	34.439	23.529	0.3568	18.063	10.28	2.602	3.0046	5.2041	354 ± 14

In contrast to the samples grown in aqueous media, in an ethanol solution, there was a clear tendency of the crystal size decrease with the deposition cycles rise up to 50. It can be explained by the inverse proportional relation between the crystal size and dislocation density referring to the formula (6). At 60 deposition cycles, the crystal size slightly increased for both cases. At the same time, larger crystal sizes in samples grown in alcohol media^44^ can be explained by the higher viscosity of ethanol ≈ 1.189 ($\eta \times {10}^{3}$(Pa*S)) compared to an aqueous solution ≈ 1.003 ($\eta \times {10}^{3}$(Pa*S))^45^. Also, particle growth is influenced by various forces, including surface tension plays an important role. Ethanol has a lower surface tension (σ = 22 mN/m) than water (σ = 71.35 mN/m). So, during the growth of ZnO with a solvent, ethanol tends to produce a more compact layer on the substrate surface and the thickness of these samples becomes thinner compared to water.

Equally important lattice parameter describing the shift of crystal structure is the micro strain (ε). ε is the root mean square of variations in the lattice parameters across the sample. Interestingly, the ε values for the ZnO-Eth samples are lower compared to ZnO-DW, which indicates the formation of highly ordered ZnO structure when ethanol was used as a solvent.

Despite the variation of SILAR cycles and use of different precursor solutions, the calculated constants of lattice (*a* = 3 Å*, c* = 5.2 Å) and interplanar distance (*d* = 2.6 Å) of the ZnO hexagonal structure were approximately the same for all samples. Based on this, it can be presumed that the use of ethanol as a solvent for ZnO does not deteriorate its crystalline basis and serves as a satisfactory alternative to a traditional aqueous solution.

### Surface morphology

**Figure 3:**
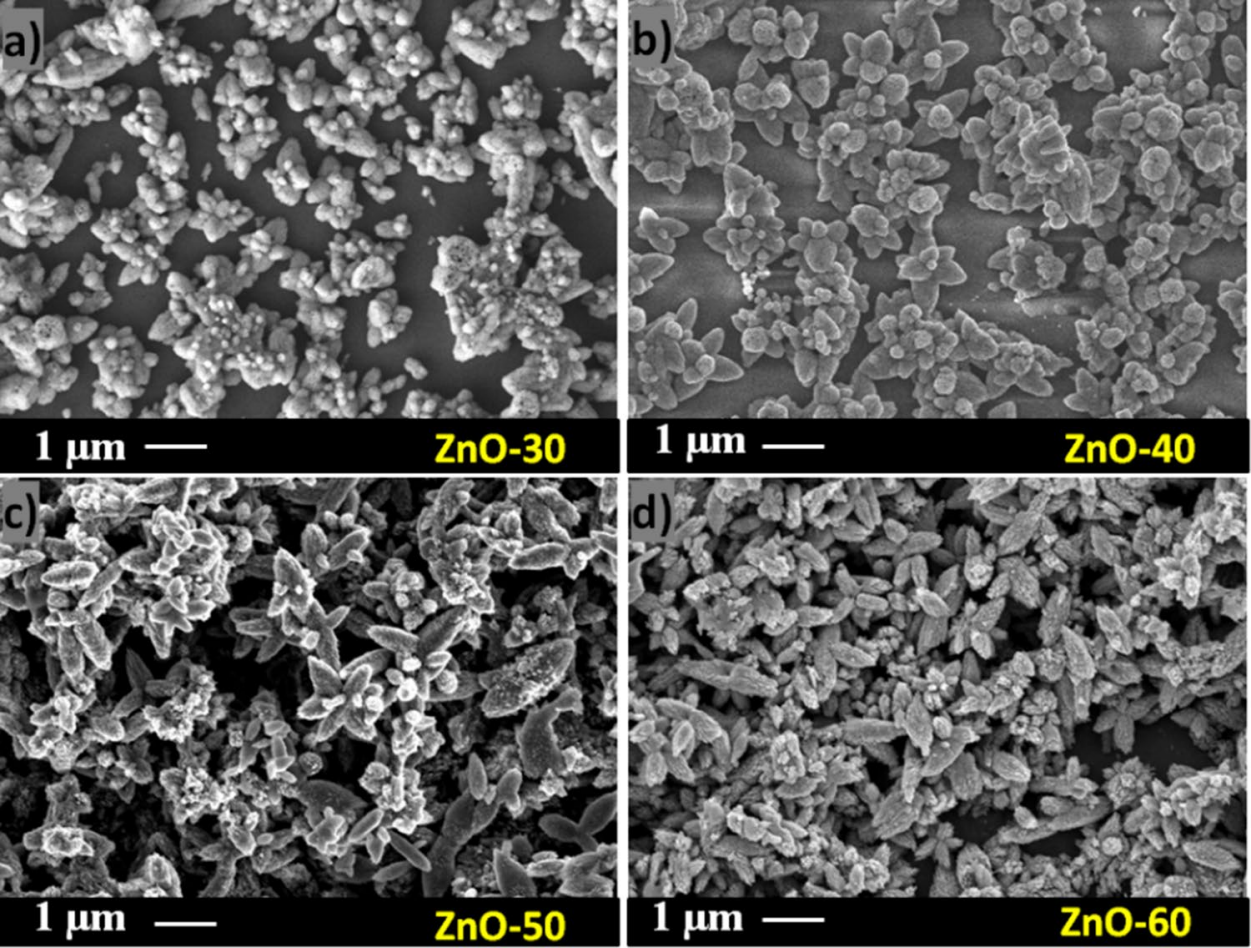
SEM images of the ZnO films grown in DW at (**a**) 30, (**b**) 40, (**c**) 50 and (**d**) 60 cycles.

Kim et al. claimed that the growth of ZnO nuclei on the substrate occurs due to the diffusion of clusters, and the fusion of these clusters leads to the formation of a homogeneous thin film^46^. As it can be seen from Fig. 3a–d that the SEM images of ZnO-DW samples have surface morphology with multiple randomly oriented structures, such as flower-like and corn-spindle. The darker areas in the SEM images represent voids with the lack of nucleation centers. In the samples ZnO-30 (Fig. 3a) and ZnO-40 (Fig. 3b), several vacuous with an inhomogeneous distribution of grains of irregular shape were observed. Despite the gradual decrease of voids within cycle number, the structure of ZnO-50 (Fig. 3c) and ZnO-60 (Fig. 3d) films and distribution of grains change chaotically. Consequently, these inhomogeneities in the structure lead to the formation of disordered layers with high roughness.

**Figure 4:**
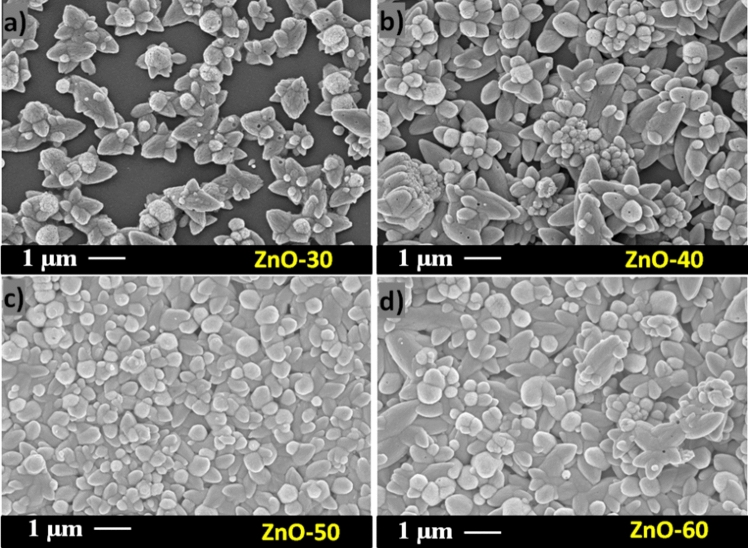
SEM images of the ZnO films grown in ethanol at (**a**) 30, (**b**) 40, (**c**) 50 and (**d**) 60 cycles.

Compared to DW samples, the morphology of ZnO-Eth samples (Fig. 4a–d) shows more compact and uniform particle structure. This is proper to the higher wettability and surpassing penetration of ethanol into mesoporous layers compared to aqueous solution^47^. In general, the clusters of particles had flower-like composition and were observed for all substrates (ethanol), despite the fact that they differ in size and number. In ZnO-30 film (Fig. 4a), it is clearly seen that particles have the flower shape and are not present everywhere. At the same time, with an increase in the deposition cycle, the surface becomes uniformity covered, which is especially noticeable for ZnO-50Eth sample (Fig. 4c). The area of the sample is well roofed with the resulting homogeneous structure. However, the sample with the higher deposition cycle as 60 (Fig. 4d), has coarser and craggier particles in the layer, which led to an increase of the surface asperity. Despite the different growth rates in both cases (DW and ethanol), an increase in the number of cycles causes the growth of layers of ZnO particles. It should be noted that, for all ZnO thin films, large defects were not observed at the multi-micron level, such as cracks or islands.

**Figure 5:**
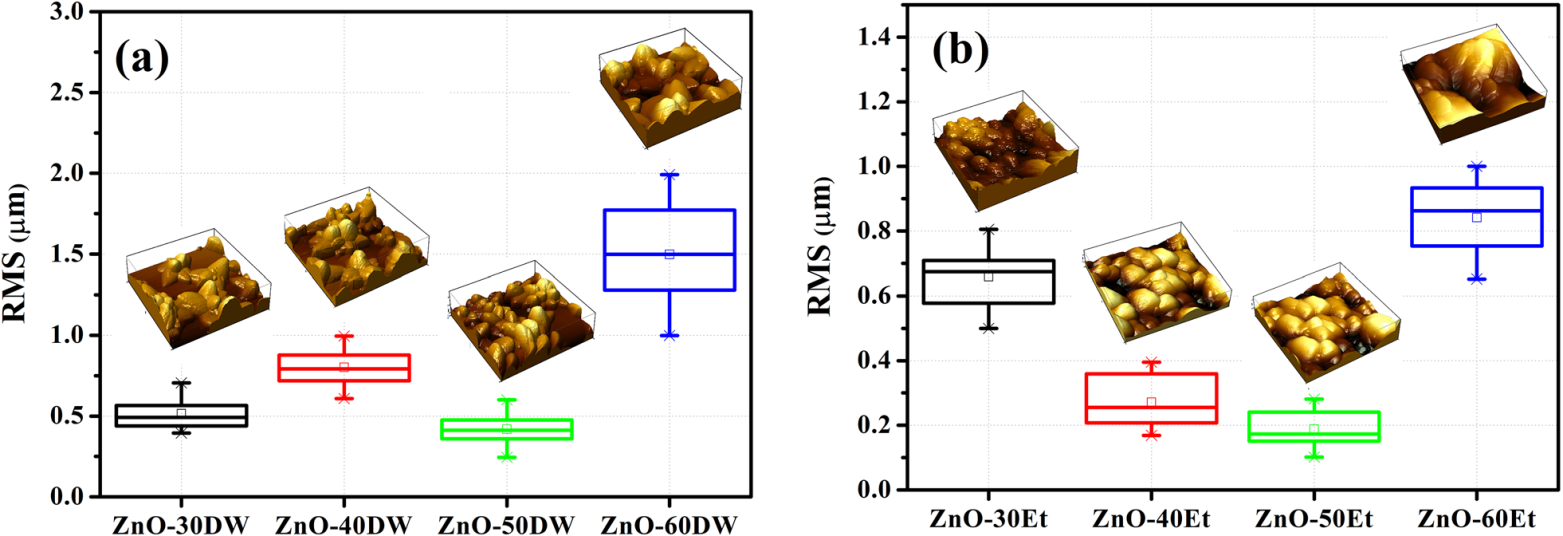
RMS values of the ZnO films grown in (**a**) DW and (**b**) ethanol solutions. Insets are corresponding AFM 3D images.

In order to study the surface morphology, the particle size distribution and asperity, AFM analysis of ZnO thin films was conducted. Figure 5a, b shows the average RMS of the thin films and insets illustrate the corresponding 3D AFM images of 5 µm × 5 µm area. ZnO thin films deposited from water solution have hillier and more cratered surface morphology. Consequently, the values of RMS show a rude spread over the deposition cycles. In contrast, ZnO thin films grown in ethanol have a smooth surface up to 50 cycles, as shown in Fig. 5b. Additionally, the particle size of ZnO-Eth samples are smaller compare to ZnO-DW (see Supplementary Information; Fig. S1). This may be due to the fact that alcohol has lower surface tension (σ = 22 mN/m) than water (σ = 71.35 mN/m)^48–50^, which leads to smoother and more uniform layer deposition on the glass substrate. So, when alcohol solvents are used, higher diffusion of ions ensures formation of sufficient nucleation centers and uniform deposition process^51^. The nucleation, diffusion and growth of ZnO nanoparticles are highly dependent on the deposition parameters (pH, time and temperature) as well as on solvent properties such as surface tension, dielectric constant and viscosity. For example, ethanol becomes more viscous at 70 °C and growth is slower and zinc oxide can be distributed uniformly over the substrate surface. Also, ZnO nanoparticles synthesized with ethanol show a smaller particle size than ZnO nanoparticles synthesized in water due to the difference in surface tension. More interesting is that nucleation and growth are accelerated if the solvent has a high dielectric constant.

The change in the roughness of the films by thickness in both cases indicates (Fig. 5a, b) an improvement of compactness of the ZnO layers up to the 50th cycle. Films of 60 deposition cycles grown in both water and ethanol showed a sharp increase of roughness. Similar trend was reported in the work of Miyata et al.^52^, explaining the behavior by increase of the crystallized regions of the film, since both crystalline and amorphous regions must coexist for the development of the texture. In general, the roughness (Fig. 5b) and thickness of ZnO films deposited in alcohol solution shown in Table 1 have better characteristics (structural and morphological) compared to the samples prepared in DW.

**Figure 6:**
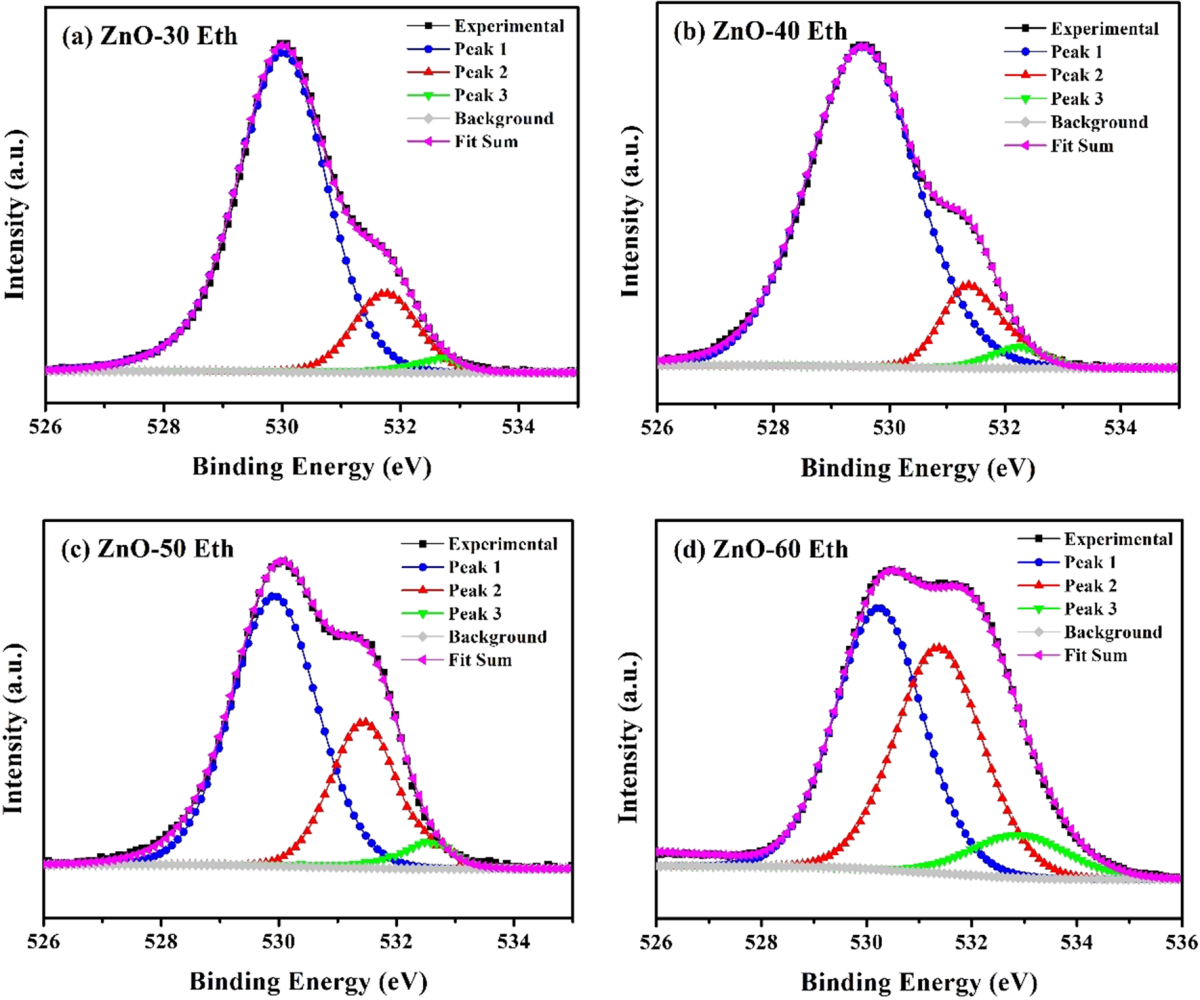
XPS O1s spectra of the (**a**) ZnO-30 Eth, (**b**) ZnO-40 Eth, (**c**) ZnO-50 Eth and (**d**) ZnO-60 Eth thin films.

XPS spectra of ZnO thin films were examined to confirm the chemical composition and energy state of the related elements. XPS survey of films indicates existence of Zn, O and C elements (see Supplementary Information; Fig. S2). The detected carbon is related to the adsorbed CO_2_ or organics on the surface during the exposure of the samples to the atmosphere. Zn2p peak arises from spin orbit splitting with binding energies of 1021.2 and 1044.3 eV, which can be ascribed to Zn-2*p*_3/2_ and Zn-2*p*_1/2_ doublets/lines (see Supplementary Information; Fig. S3). The binding energy difference between two lines is $\sim$ 23.0 eV, which is comfortably lying close to the standard reference value of ZnO^53^. The values of the binding energy and binding energy difference, obtained from the XPS, show that the Zn atoms are in the Zn^2+^ oxidation state^54^. In general, the carrier transport properties in ZnO are closely related to the chemical states of the elements in the ZnO, such as the metal–oxygen bond, oxygen vacancies (V_o_), and hydroxyl groups (OH). O1*s* peak of ZnO thin films grown in ethanol is shown in Fig. 6a–d. O1*s* peak can be deconvoluted into three peaks. The first peak represents O^2−^ ions in a wurtzite structure surrounded by Zn atoms at the low binding energy ($\sim$ 529.9 eV); the second peak represented the oxygen vacancies at medium binding energy ($\sim$ 531.5 eV); and third peak represented OH species and/or aqueous adsorbed on the surface of ZnO film at a high binding energy ($\sim$ 532.9 eV)^55^. It was found that with an increase in the deposition cycle, the metal–oxygen bond is weakened, while the intensities of V_o_ and the OH group raised. However, the O1*s* peak (Peak 3) of ZnO-Eth is lower compare to the samples grown in water (see Supplementary Information; Fig. S4). Thus, it can be assumed that oxygen vacancies are limited in ZnO-Eth samples, which indicates a decrease in point defects.

### Optical and electrical properties

**Figure 7:**
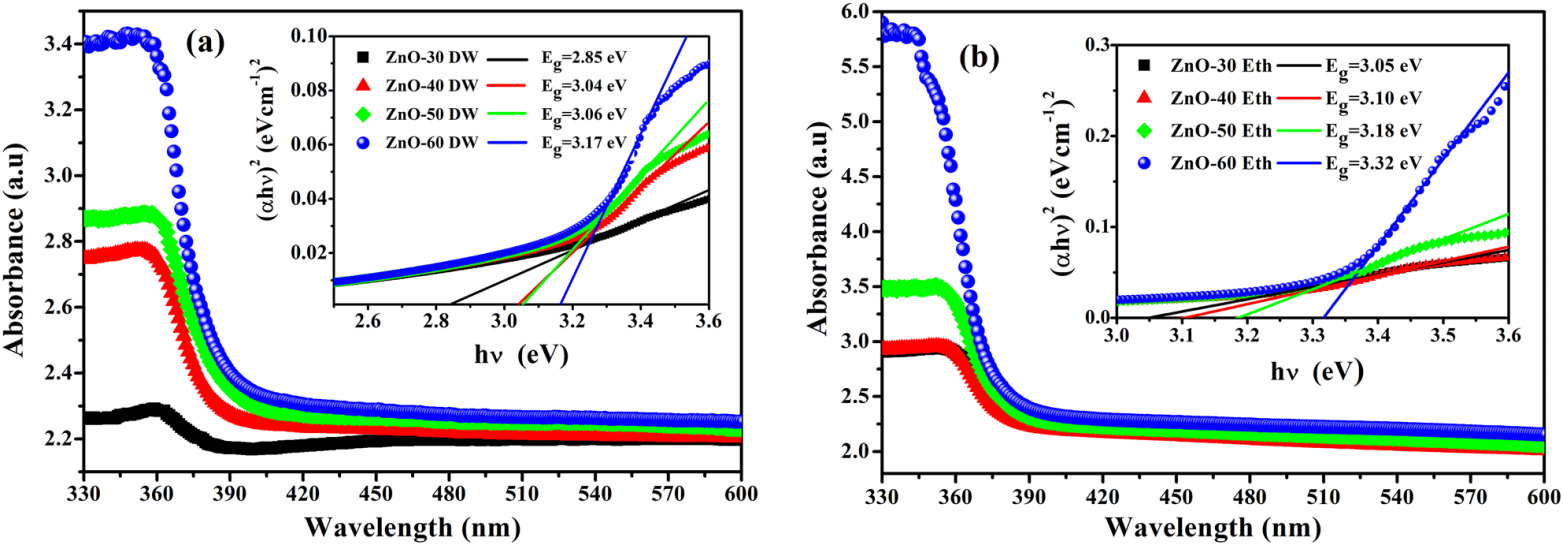
UV absorption and (*αhν*)^2^ versus photon energy (*hν*) plots of ZnO films grown in (**a**) DW and (**b**) ethanol solvents.

Electro-optical studies of metal oxide thin films provide quite essential information regarding physical properties, such as optically active defects, band structure the and bandgap energy^56^. The influence of thickness and the solvent precursor on the values of the band gap energy (E_g_) and the optical absorption of metal oxide thin films have been also investigated. UV spectroscopy measurements were fulfilled with a UV–Vis spectrometer between wavelengths of 330 nm and 600 nm. UV absorption and (*αhν*)^2^ vs. photon energy (*hν)* plots of the ZnO thin films grown by DW and ethanol solvent are depicted in Fig. 7a, b. As shown in Fig. 7a, b, all thin films grown in DW and ethanol solvent have low absorbance in the infrared area and high absorbance in the UV area. These results are in good agreement with other studies^25,57^. Also, it was found that with an increase in thickness and roughness, the absorption of a thin film increased, since with an increase in film roughness, more and more photons can be adsorbed on the surface of the material. In addition, it can be seen from the absorption spectra that the absorption edge is directly proportional to the thickness value and shifts towards higher energies. E_g_ of thin films can be defined by the well-known Tauc formula^58^. According to this formula, the relation between E_g_ and the absorption coefficient is given by using the following expression:

9 $$\alpha =\frac{A}{hv}{\left(hv-{E}_{g}\right)}^\frac{1}{2}$$

here, *A* is a constant, *h* is Planck’s constant,* α* is the absorption coefficient, *ν* is the frequency of photon and E_g_ is the optical bandgap energy of thin films. The obtained bandgap energies of thin films are given inset Fig. 7. The calculated E_g_ for ZnO thin films grown using DW can vary from 2.85 to 3.17 eV, while the E_g_ for ZnO thin films grown using ethanol solvent can vary from 3.05 to 3.32 eV, respectively. The bandgap energy values slightly increase with the ZnO thin film thickness in both cases. The increment of bandgap energy is explained that during the growth of ZnO nanostructures lattice deformation of the film decreases^59^. Besides, electrons in the metal oxide semiconductor experience the periodic potential of the ZnO crystal lattice. This potential leads to the increasing of the bandgap energy. Additionally, a change in the bandgap is associated with a change in the dislocation density, lattice strain, and the formation of defects in the ZnO^60^. Ilican et al., reported that the lattice strain affected on bandgap energy of ZnO nanostructure by changing the interatomic spacing^61^ and E_g_ values increment for an increase in strain along the c-axis but decrement for an increase in a tensile strain of ZnO structure^62^. Ansari et al. studied the bandgap narrowing of the ZnO nanostructure caused by oxygen vacancies^63^. They explained that the ZnO grown in water has more vacancies of oxides which leads to a decrease in the bandgap of ZnO. When the film thickness changed from 136 to 354 nm (ethanol media), a slight change in the bandgap energy of ZnO thin were observed. The optical measurements result suggests that E_g_ of ZnO thin films alters insignificantly by the thickness and roughness changes, but rather controlled by the structural features, which in turn depend on the nature of precursor solvent, as well as a reaction media.

**Figure 8:**
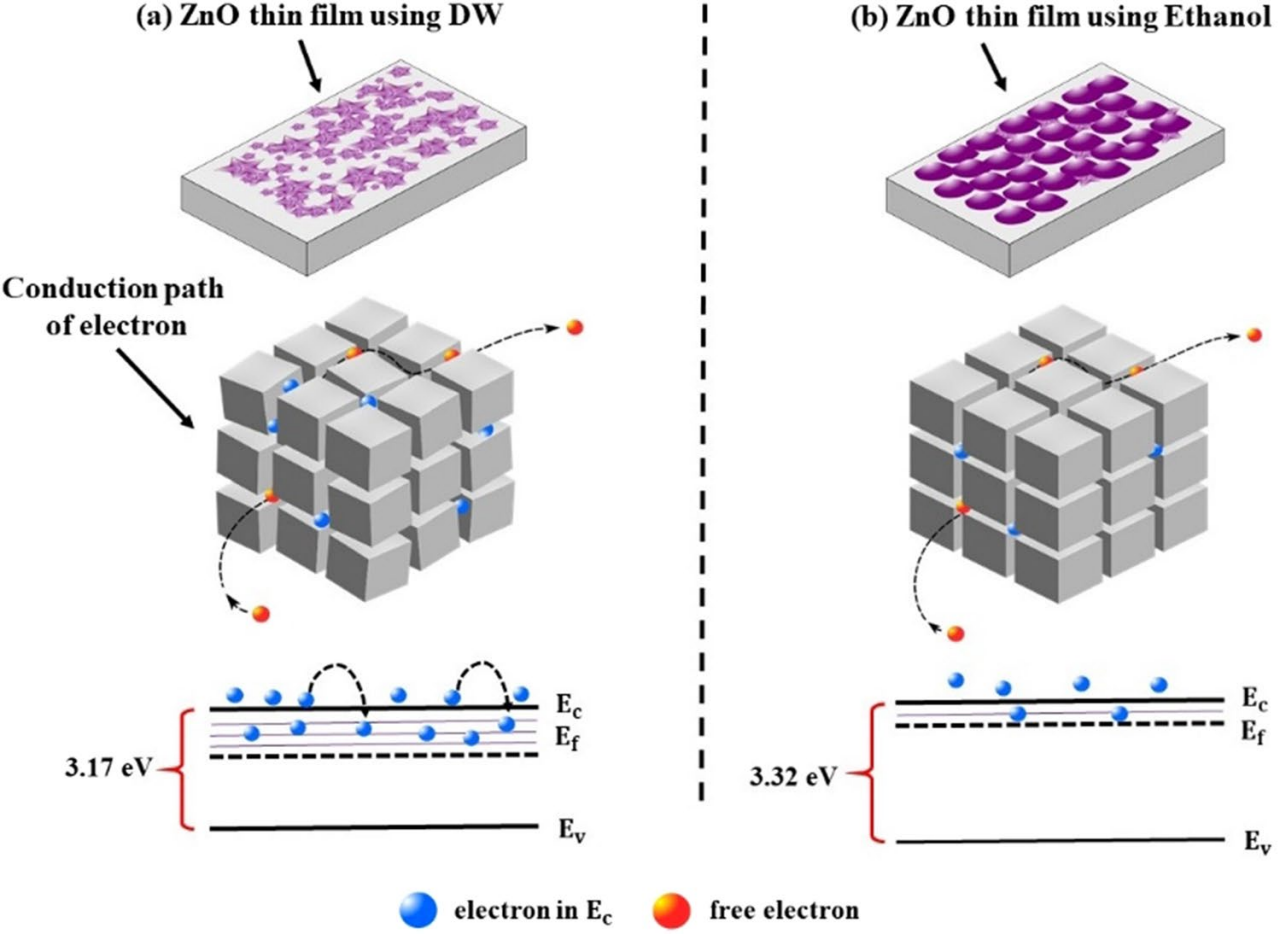
Illustration of conduction pathway of an electron at grains and grain boundary in ZnO films grown using (**a**) DW and (**b**) ethanol.

Besides, the results of the optical measurements indicate that ZnO thin films grown with ethanol have higher bandgap energy values compared with the thin films grown with DW. The expansion of band gaps in ZnO thin films grown with ethanol can be explained by the Burstein–Moss effect^64^, which came out due to the increase of carrier concentration. As seen in Fig. 8, random oriented grains have the property of a rougher surface; while grains are well aligned along the c-axis have a smooth surface. In ZnO nano-crystalline structure, the electron trapping sites at grain boundaries control electron conduction^35,65–67^.

Figure 8 depicted the conduction pathway of electrons in grains and at grain boundaries for erratically oriented grains in ZnO thin film grown using DW (Fig. 8a) and for the evenly oriented grains for the ZnO thin film grown using ethanol (Fig. 8a). The ZnO thin film grown using ethanol has fewer defects and capture sites. The bandgap energy schemas of Fig. 8b show the depletion of the trapping state density for the ZnO thin film grown using ethanol. Thereby, it can be assumed that the Fermi level shifts to a higher energy level above the minimum of the conduction band, which constructively expands the bandgap energy of the ZnO^68^.

**Figure 9:**
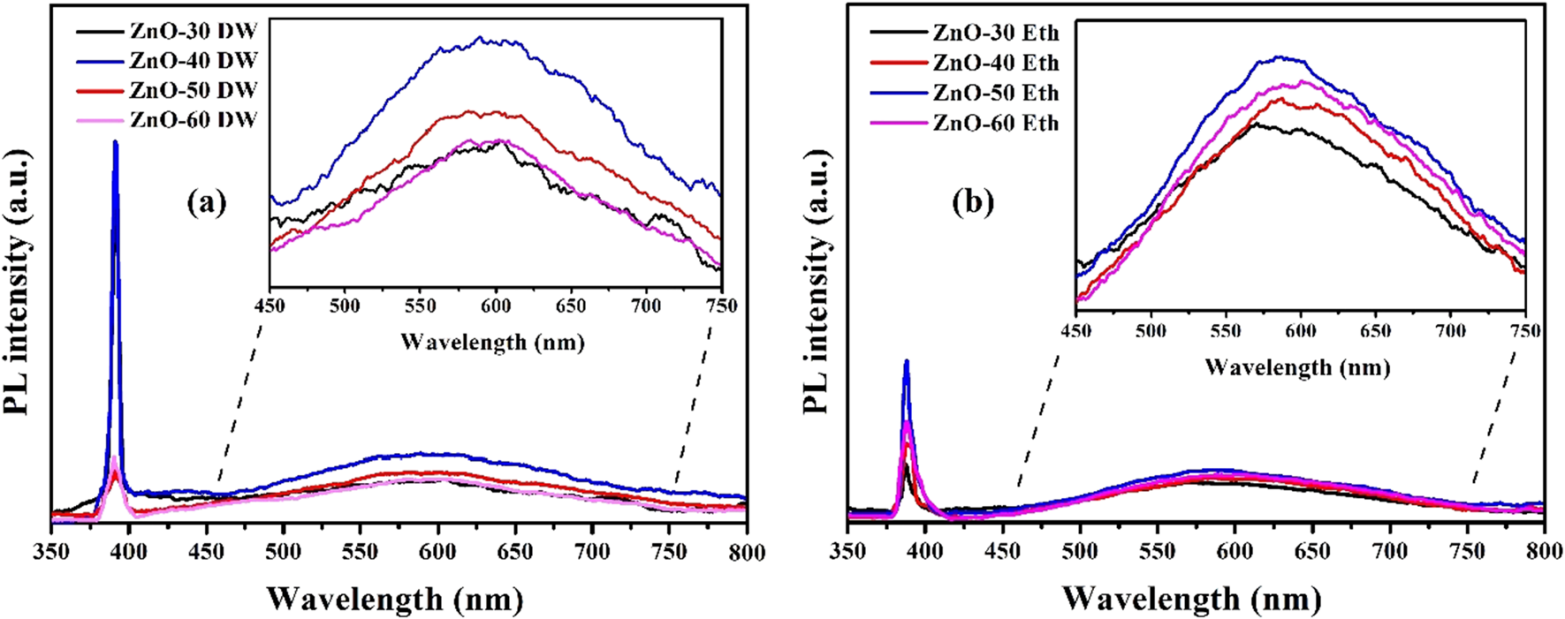
PL spectra of ZnO thin films grown in (**a**) DW and (**b**) ethanol.

PL spectroscopy results demonstrated that the ZnO exhibited two peaks, one is in the UV range and another is in the visible range. As shown in Fig. 9a, the ZnO-DW have the strongest a near-band-edge (NBE) emission at the UV region centered at about 379 nm. Among the ZnO films deposited from water solution, ZnO-40 DW sample release a strong intensity emission in the NBE region^69^. A broad deep-level emission near the green light (560 nm) is a second peak which apparently to be intrinsic in ZnO^70^. The emission peak at 560 nm was observed in the ZnO-Eth (Fig. 9b) and ZnO-DW samples and might be attributed to the presence of oxygen vacancies (V_o_) and zinc interstitial (Zn_i_), which can be formed in pure ZnO^71^. Figure 9a, b (inset) clearly shows that when the deposition cycle was increased to 50, the intensity of the green emission peaks was reduced in both samples (ZnO-DW and ZnO-Eth). Thus, we can assume that the intensity of the green emission peaks may depend on the film thickness. Also, the intensity of the green emission peaks for the ZnO-50 DW sample slightly increased compared to the ZnO-50 Eth sample (see Supplementary Information; Fig. S5). Furthermore, UV–Vis spectrophotometer was used to determine the optical transmittance spectra of ZnO films in a wavelength range of 300–800 nm (see Supplementary Information; Fig. S6). The transmittance of the ZnO-Eth appeared to be higher than the ZnO-DW, for example for ZnO-40 Eth and ZnO-40 DW samples show $27.54$ and $5.40 \%$, respectively. The phenomenon of increased visible-range transmittance of ZnO-40 Eth can be explained by the reduction in oxygen vacancies which can lead to a decrease in light absorption and an increase in light transmittance in the visible light region^72^.

**Figure 10:**
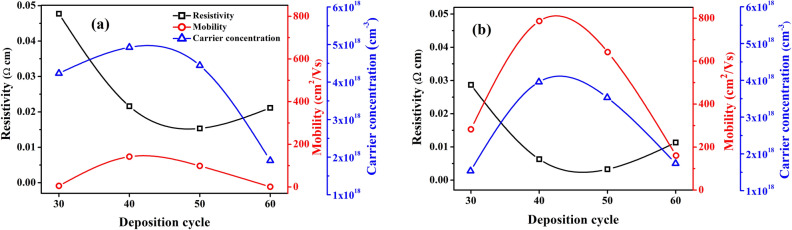
Hall mobility, Resistivity and electron carrier concentration of ZnO films synthesized using (**a**) DW and (**b**) ethanol solvents.

The detailed study of the electrical features of ZnO films were carried out by HMS-5500. The resistivity, carrier concentration and mobility of ZnO thin films grown using DW and ethanol as precursor solvents are shown in Fig. 10a, b. Higher carrier mobility in ZnO-Eth thin films (Fig. 10b) might be associated with decrease in the carrier concentration. The carrier mobility increases result in a substantial lower probability of mutual scattering in the structure of the ZnO lattice^73^. For the ZnO-DW thin films, the carrier concentration increases within 40 cycles, and drops by ZnO-60. The same trend was observed for ZnO-Eth thin films. This phenomenon is due to the fact that with an increase in the deposition cycle more defects are formed which capture electrons and prevent the movement of carriers. Besides, the temperature-dependent mobility of ZnO films was measured from room temperature to 700 K. In general, the grain boundary scattering is dominant in ZnO compared to other scattering mechanisms^74^. It is clearly seen that the mobility of ZnO thin film grown in ethanol is larger than that of ZnO thin film grown in DW due to the better surface and textured structure (see Supplementary Information; Fig. S7). The improvement in mobility can be attributed to a decline in dislocation density and lattice strain^75^.

### Photosensitive properties of the ZnO UV sensor

**Figure 11:**
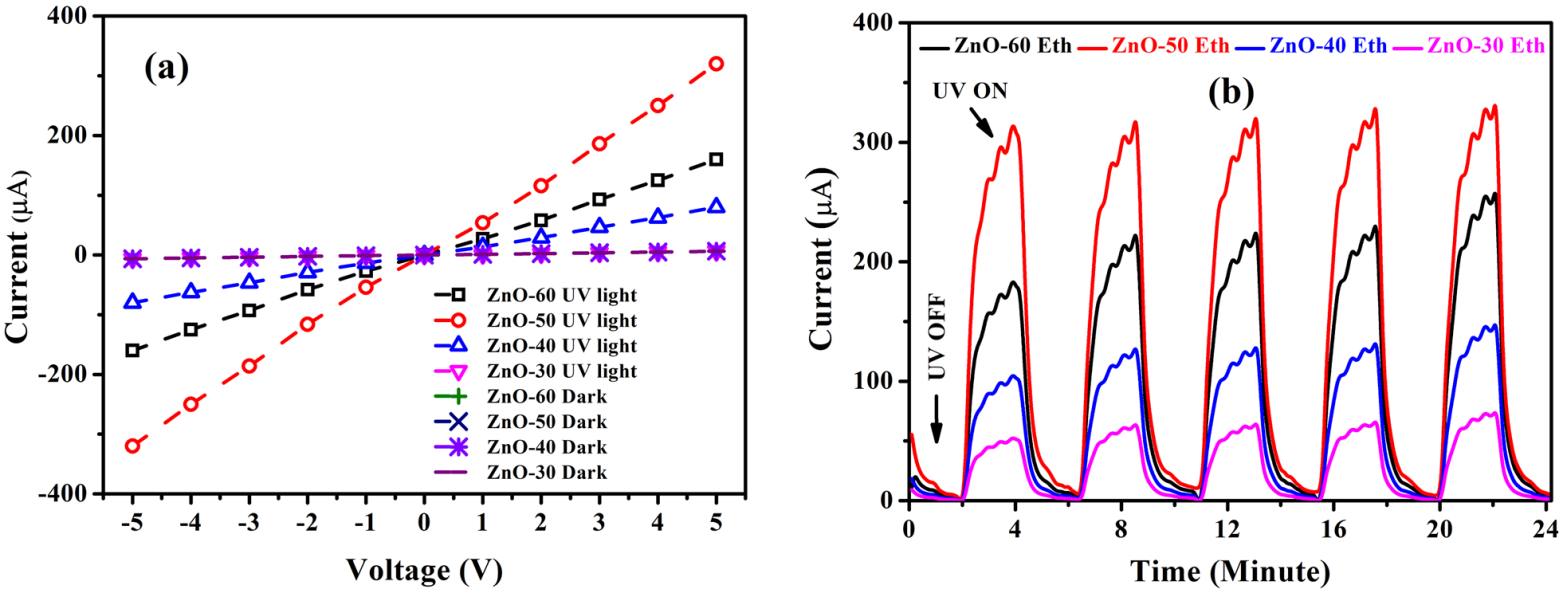
(**a**) I–V characteristics and (**b**) UV response of the ZnO thin films grown in ethanol as a function of time upon 2 mW/cm^2^ illuminations at 5 V voltage.

To investigate the photosensitive properties of the ZnO films, the current–voltage (I–V) characteristics and UV response were studied under the dark and UV light illuminations. The fabrication of UV sensor was conducted by magnetron sputtering the electrodes on the surface of ZnO (see Supplementary Information; Fig. S8). As shown in Fig. 11a, IV characteristics of ZnO UV sensor is nearly symmetric as well as linear with forward and reverse bias. This result indicate the formation of ohmic contacts between Au electrodes and a photosensitive ZnO film at a bias voltage of up to 5 V. Such ohmic contacts play an important role in the photosensitive properties of the UV sensor, which can be maximized when the semiconductor–metal junction is an ohmic contact^76–78^. The maximum photocurrent (UV current) was around 324 μA at 5 V bias voltage for ZnO-50 Eth. Figure 11b shows the on–off switching UV response of the ZnO-Eth. The photocurrent shows an increasing value upon exposure to UV illumination, and the current decreases exponentially under dark conditions. In dark conditions, the oxygen molecules are adsorbed onto the surface of the ZnO and capture free electrons in the valance band to the conduction band, and form O^-^ and O^2-^ which creates a depletion region (high resistance area) near the ZnO surface. Under UV light illumination at photon energies (hv) above the bandgap energy of the ZnO, electron–hole pairs are generated.

The holes migrate to the ZnO surface and getneutralized by the negatively charged surface oxygens (O^–^ and O^2–^). This process leads to producing the unpaired electrons, which serve as the majority charge carriers and contribute to the increase in the conductivity under an applied electric field^79^. According to the UV response results, the ZnO films grown in ethanol have high UV responses, and the reproducibility was quite stable compared to the ZnO films grown in water (see Supplementary Information; Fig. S9). Also, compared to other works^80–83^, our prepared UV sensor depicted relatively high UV response and excellent repeatability, which are attributed to the high crystal quality and fewer defects in ZnO.

## Conclusions

In summary, ZnO thin films of different thickness were synthesized on glass substrates by the SILAR method using distilled water and ethanol as precursor solvents. ZnO thin films grown using ethanol are a high crystalline structure compared to the films grown using distilled water. Nano-flower morphology was observed for ZnO films grown using distilled water and ethanol while the surfaces of films grown with high SILAR cycle number are covered with nanostructures. Interestingly, for the samples grown in ethanol solvent the grain size decrease from ~ 24 to ~ 19 nm and was observed by an increase of the SILAR deposition cycles up to 50. The samples demonstrated higher bandgap energy with regard to the films grown using distilled water owing to a high crystallinity and fewer defect formed within the bandgap. The highest carrier mobility was obtained for ZnO-Eth films. Also, ZnO-Eth films showed high UV responses and the repeatability was stable under UV illumination. Thereby, we have concluded that using ethanol as a solvent for precursors in the SILAR method can provide high-quality ZnO films, which are beneficial in applications for UV photodetector and dye-sensitized solar cells, etc. It also widens the choice of precursors and enables the use of precursor’s which insoluble in water. Further work will be dedicated to exploring the gas-sensing and dye-sensitized performance of ZnO thin films.

## Supplementary Information

Supplementary Information.

## Data Availability

All data supporting the conclusions of this article are included within the article.

## Abbreviations

AFM: Atomic force microscopy
BE: Binding energy
CB: Conduction band
DW: Distilled water
E_g_: Band gap energy
FWHM: Full width at half maximum
RMS: Root mean square
SEM: Scanning electron microscopy
SILAR: Successive ionic layer adsorption and reaction
UV–Vis: Ultraviolet–visible spectrophotometry
XRD: X-ray diffraction
ZnO: Zinc oxide
