# Supplementary material for: Effect of thickness and reaction media on properties of ZnO thin films by SILAR
Source: Sci Rep. 2022 Jan 17;12:851. doi: 10.1038/s41598-022-04782-2 (PMC8764087; doi:10.1038/s41598-022-04782-2)
Supplement: Supplementary file 1 — Supplementary Information. [file 41598_2022_4782_MOESM1_ESM.docx]

***Supporting Information***

**Effect of Thickness and Reaction Media on Properties of ZnO Thin Films by SILAR**

Gani Yergaliuly^1,2^, Baktiyar Soltabayev^1,3*^, Sandugash Kalybekkyzy^1,3^, Zhumabay Bakenov^1,3^, Almagul Mentbayeva^1,3*^

^1^Department of Chemical and Material Engineering, School of Engineering and Digital Sciences, Nazarbayev University, Nur-Sultan 010000, Kazakhstan

^2^L.N. Gumilyov Eurasian National University, Nur-Sultan 010000, Kazakhstan

^3^National Laboratory Astana, Nazarbayev University, Nur-Sultan 010000, Kazakhstan

*Corresponding authors:* [baktiyar.soltabayev@nu.edu.kz](mailto:baktiyar.soltabayev@nu.edu.kz) (B. Soltabayev), [almagul.mentbayeva@nu.edu.kz](mailto:almagul.mentbayeva@nu.edu.kz) (A. Mentbayeva)

**A. The particle size of films calculated by AFM images for ZnO thin films**

**
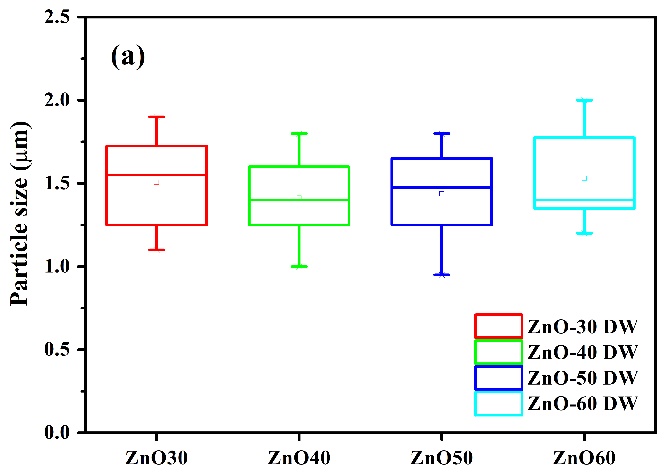

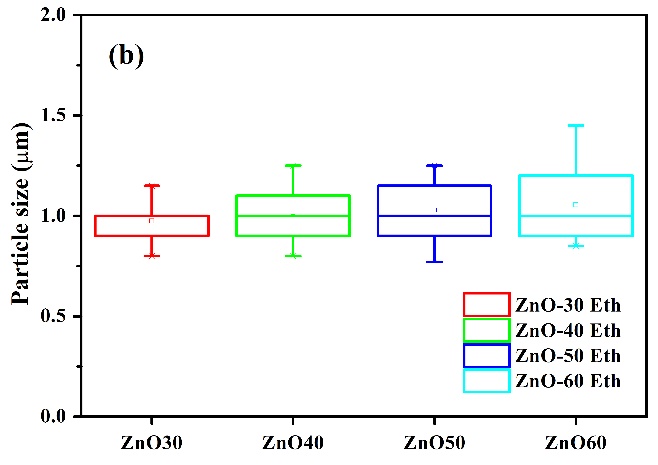
**

**Fig. S1.** The particle size of films calculated by AFM images for ZnO thin films grown in (a) DW (b) ethanol

**B. XPS spectrums of ZnO thin film grown in DW and ethanol**


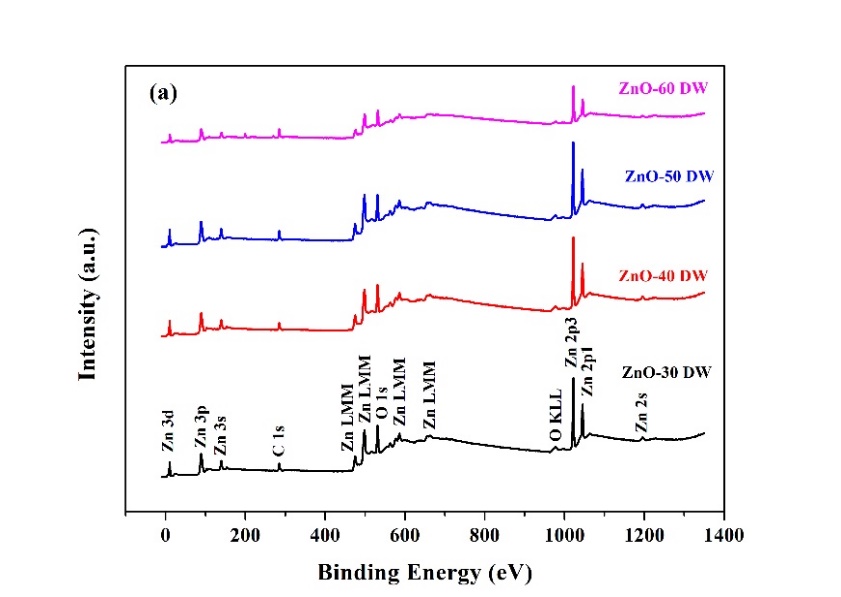

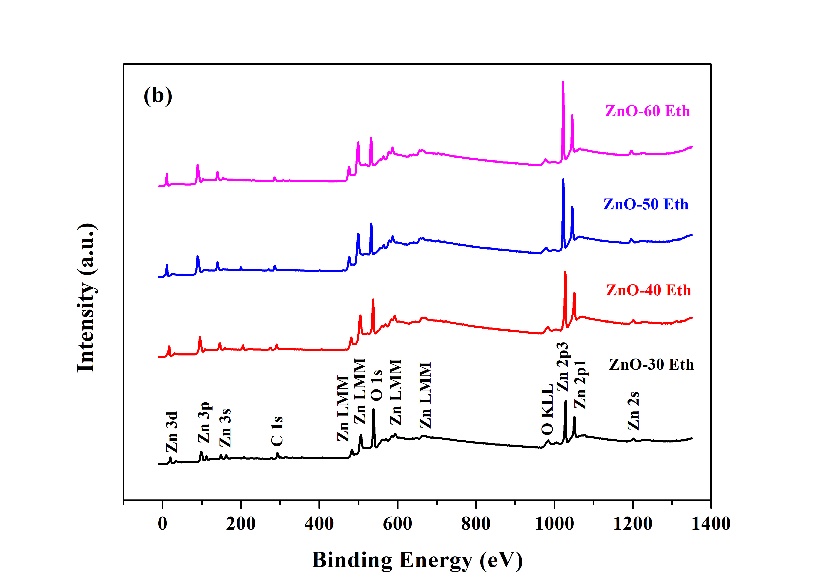


**Fig. S2.** XPS Survey of a) ZnO thin films grown in DW and b) ZnO thin films grown in ethanol


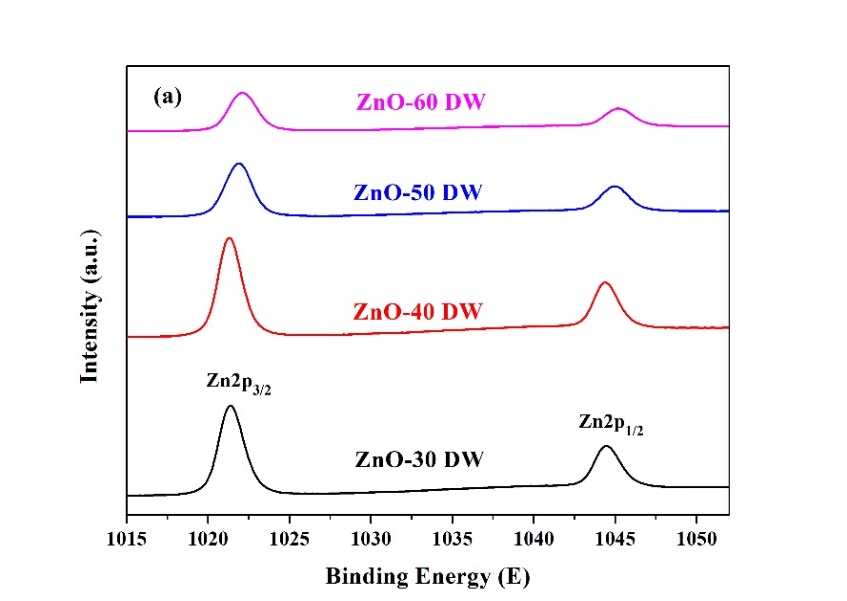

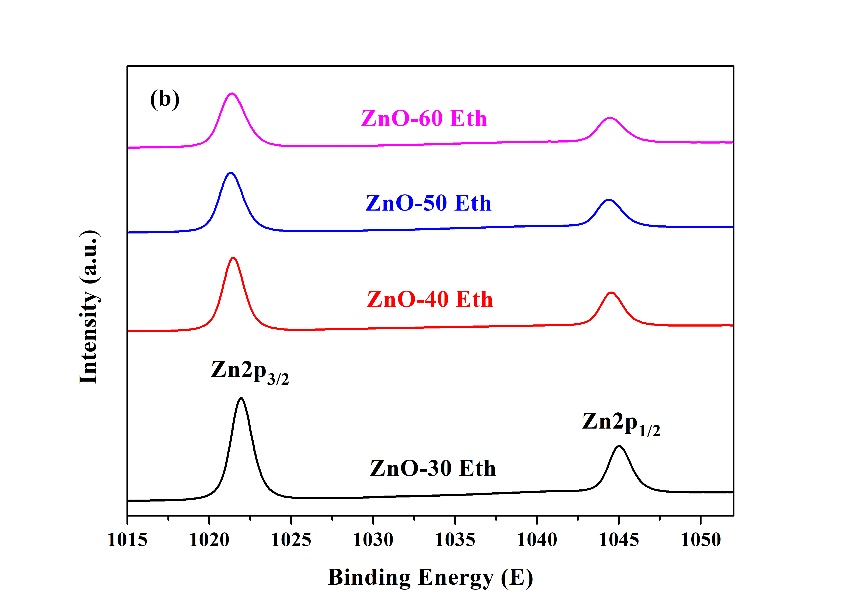


**Fig. S3.** XPS Survey of a) ZnO thin films grown in DW and b) ZnO thin films grown in ethanol

**
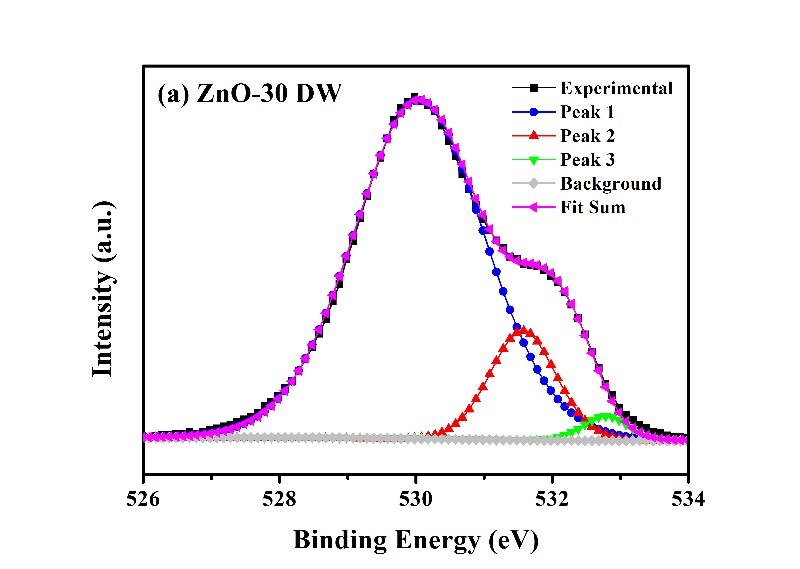

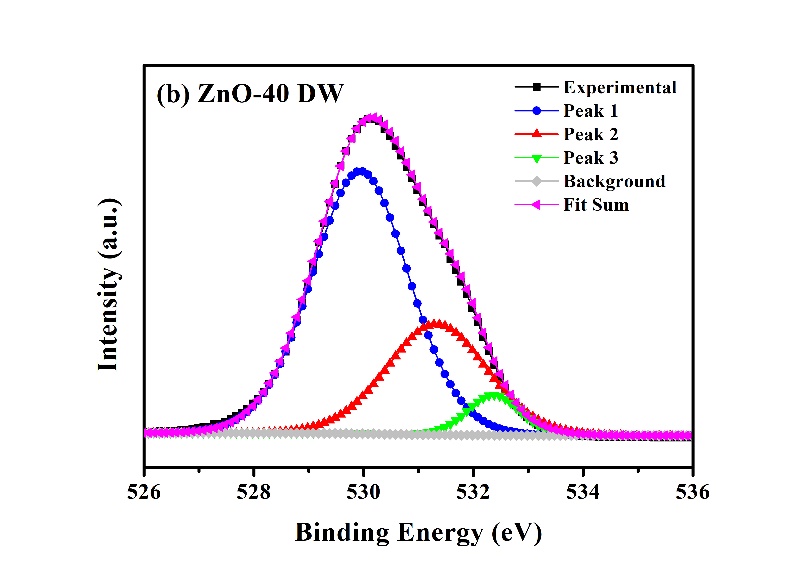
**

**
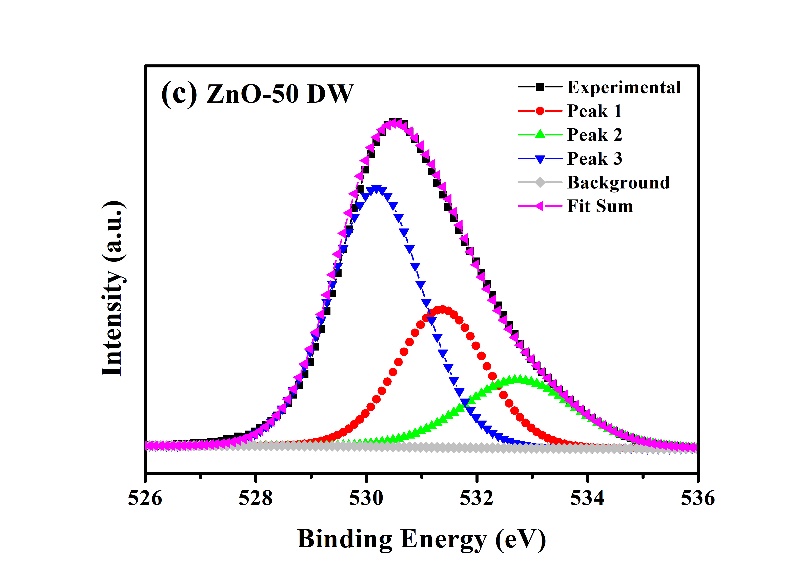

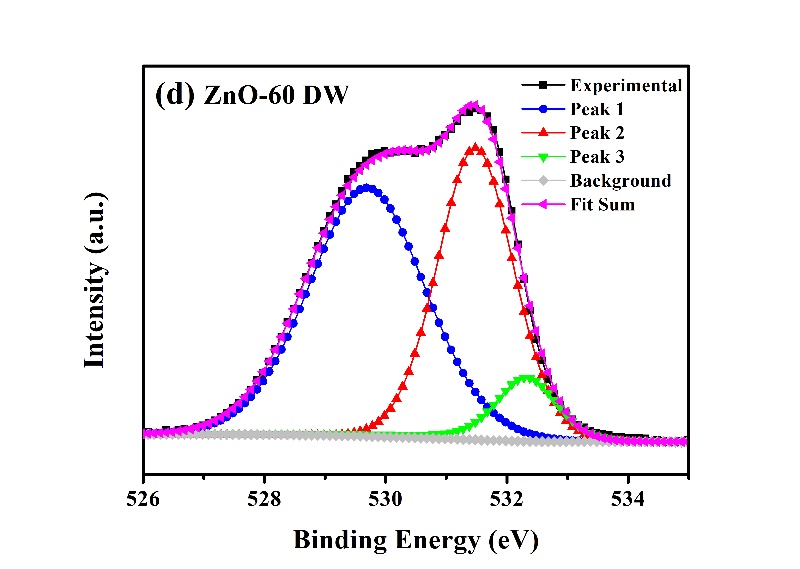
**

**Fig. S4.** XPS O1s spectra of the (a) ZnO-30 DW, (b) ZnO-40 DW, (c) ZnO-50 DW and (d) ZnO-60 DW thin films

**C. PL spectra of ZnO-50 DW and ZnO-50 Ethanol**


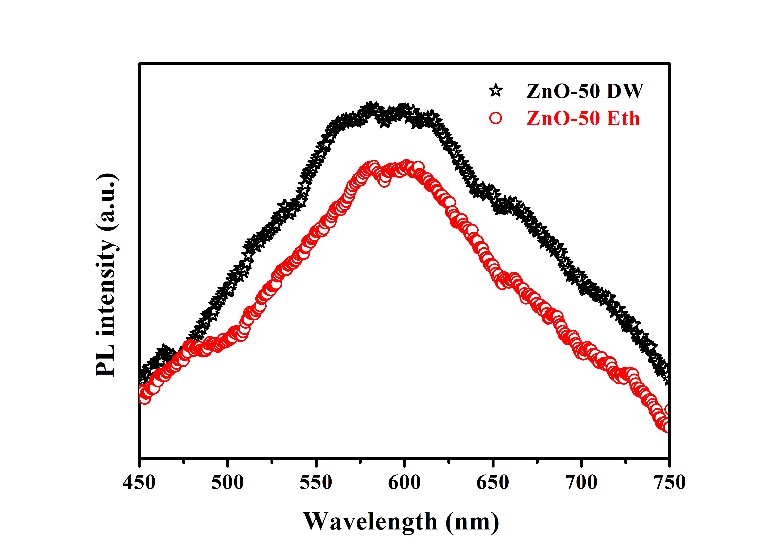


**Fig. S5.** PL spectra of ZnO-50 DW and ZnO-50 Eth

**D. UV–vis transmission of the ZnO films grown in DW and ethanol**


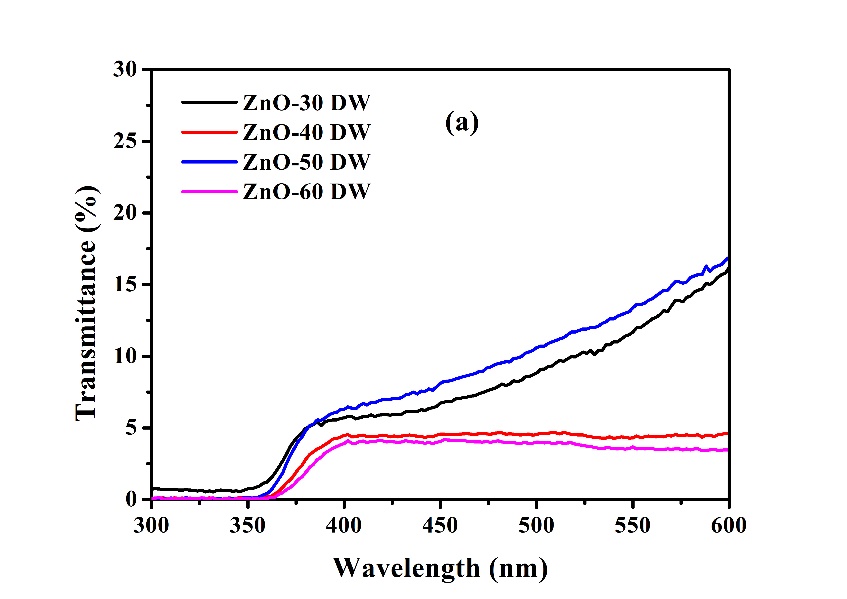

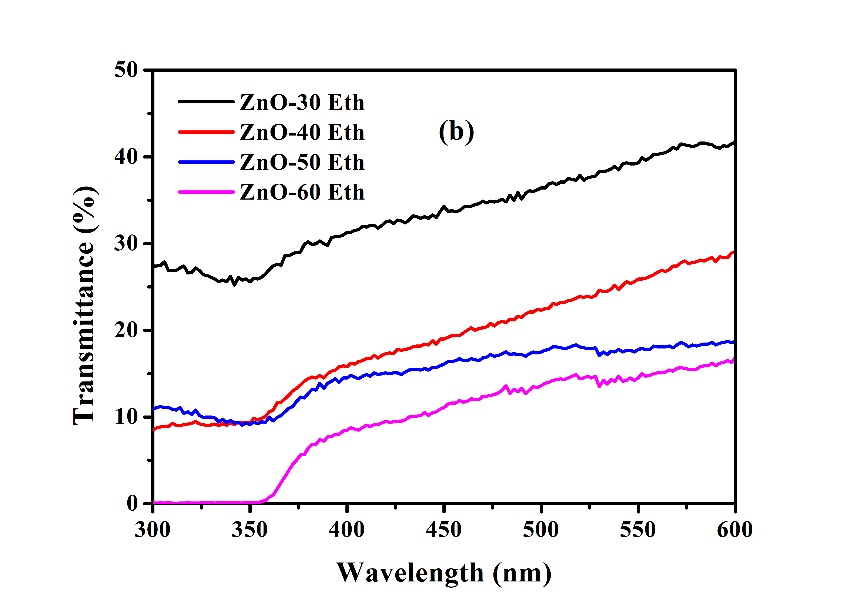


**Fig. S6.** UV–vis transmissions of the ZnO thin films grown in (a) DW and (b) ethanol as a function of wavelength in the range of 300 to 800 nm at room temperature.

**E. The temperature-dependent electron mobility of the ZnO films grown in DW and ethanol**

The temperature-dependent electron mobility of the ZnO films is shown in Fig. S7 In a room temperature (300 K), the slopes of mobility vs. temperature (T) curves of ZnO thin film grown in DW (Fig. S8-a) and ethanol (Fig. S8-a) are very similar, which means that the electron mobility is determined by the grain boundary scattering.


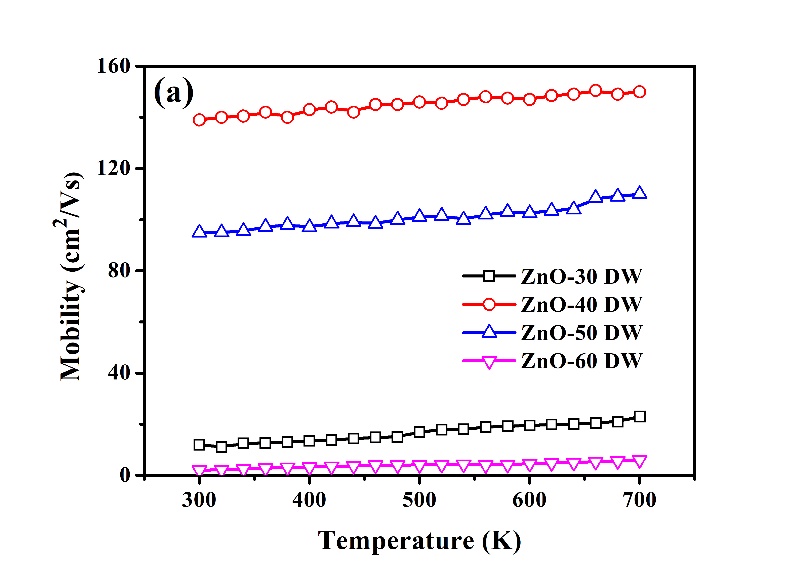

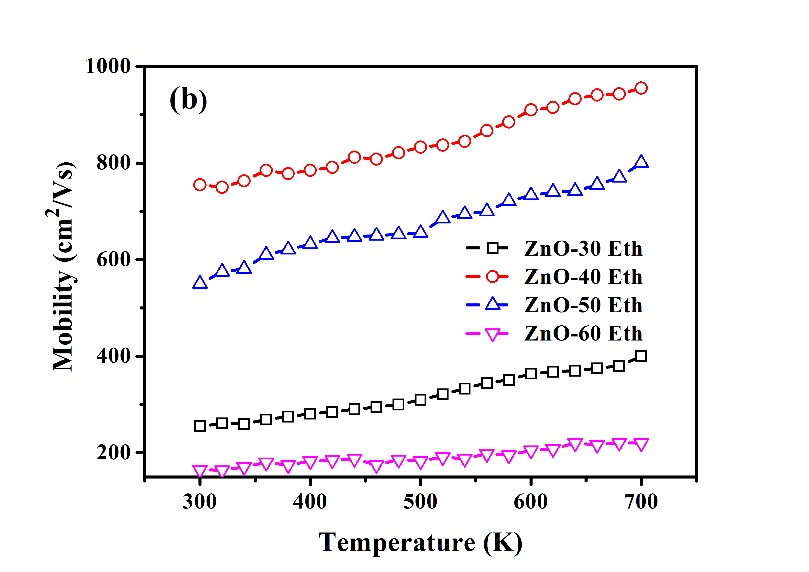


**Fig. S7.** Hall mobility of ZnO thin films grown in DW (a) and ethanol (b) as a function of deposition temperature in the range of 300 to 700 K.

**F. Fabrication of ZnO UV sensor**

The fabrication of the ZnO-based UV sensor was conducted by sputtering gold (Au) grid contact with 800 nm thickness on top of the ZnO layer by using an aluminum metal mask, as shown in Fig. S8. The structure of contact metal Au consisted of two interdigitated contacts (electrode) with four rods each. Each rods has a length of 3.1 mm, a width of 0.7 mm, and a spacing of 300 μm between the width and the length. The active area of the ZnO-structured UV sensor was 0.3 cm^2^. Au grid was deposited using DC magnetron sputtering with a DC power of 240W and sputter pressure 5 mTorr/20sccm. High-purity argon gas was used as sputtering gas at a fixed ratio of 20%. The interdigital transducer electrode (IDT) was deposited at room temperature and without substrate heating.


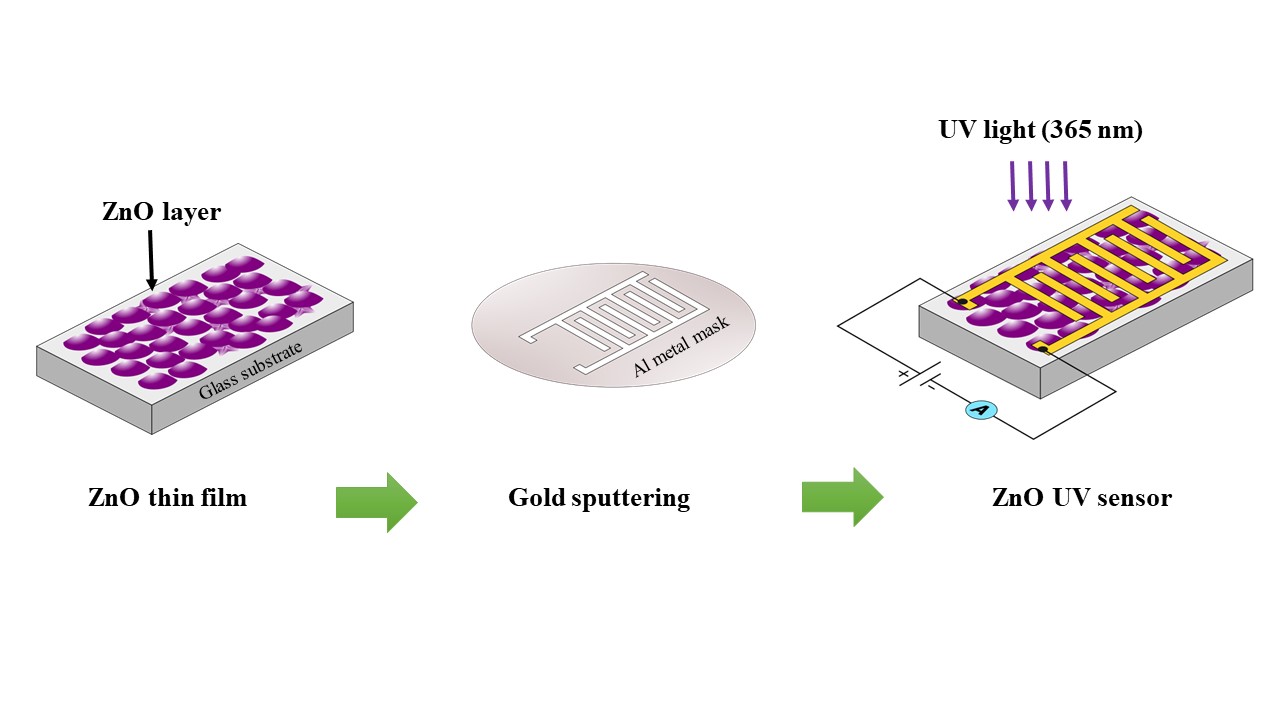


**Fig. S8.** Schematic illustration of the preparation of ZnO UV sensor

**G. I-V characteristic and UV response of the ZnO DW**

Fig. S9(a) shows I-V characteristics of the ZnO thin films grown in DW measured in dark and under UV-light illumination conditions. The photocurrent was measured at the same illumination power (2 mW/cm^2^) for the ZnO thin films grown in DW and ethanol. The photocurrent measurements were performed by varying the voltages (from +5V to -5V). Fig. S9(b) shows on–off switching characteristics of the ZnO DW with a pulsed UV (365 nm) under 2 mW/cm^2^ UV illumination at 5 V voltage. The on and off time durations are both 2 minutes.


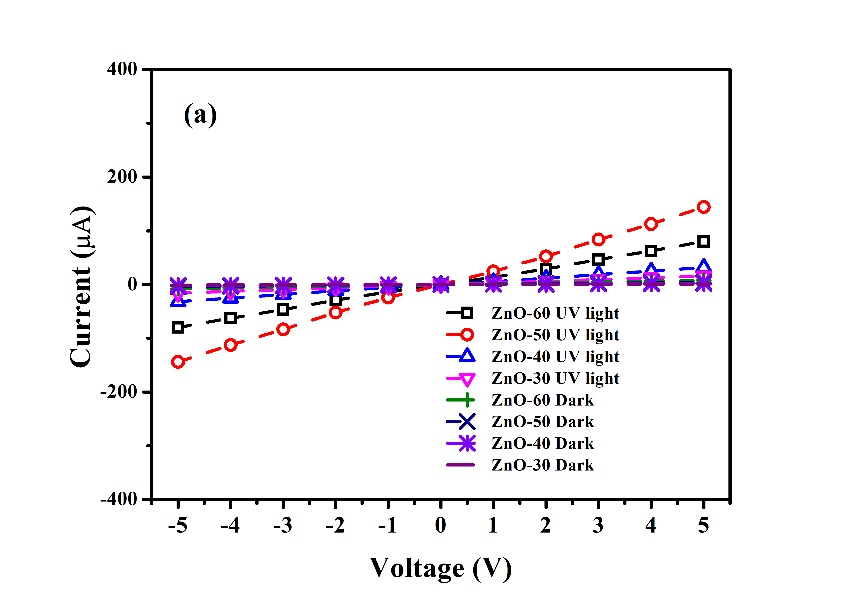

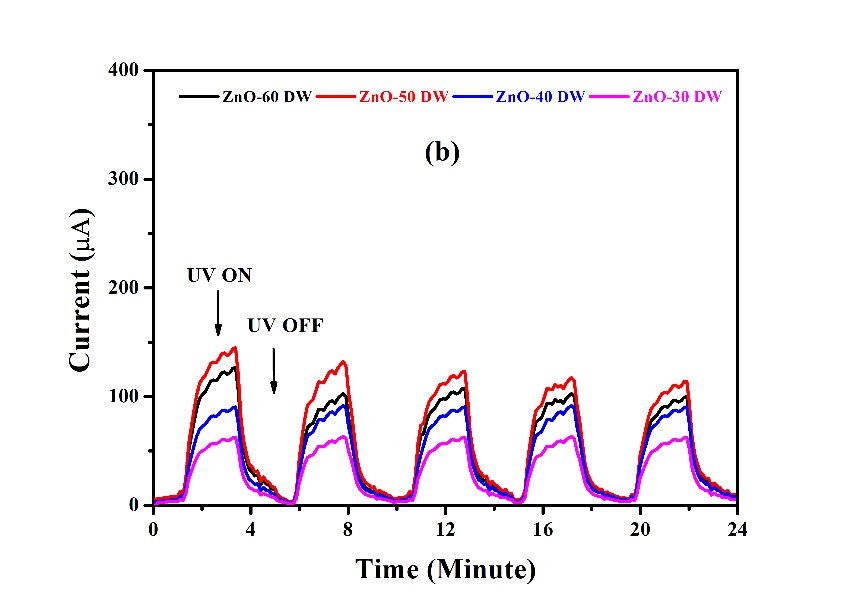


**Fig. S9.** (a) I-V characteristics and (b) UV response of the ZnO thin films grown in DW as a function of time upon 2 mW/cm^2^ illuminations at 5 V voltage.
